# ER residency of the ceramide phosphoethanolamine synthase SMSr relies on homotypic oligomerization mediated by its SAM domain

**DOI:** 10.1038/srep41290

**Published:** 2017-01-25

**Authors:** Birol Cabukusta, Matthijs Kol, Laura Kneller, Angelika Hilderink, Andreas Bickert, John G. M. Mina, Sergei Korneev, Joost C. M. Holthuis

**Affiliations:** 1 Molecular Cell Biology Division, Faculty of Biology/Chemistry, University of Osnabrück, 49076 Osnabrück, Germany; 2 Molecular Genetics, Life and Medical Sciences Institute, University of Bonn, 53115 Bonn, Germany

## Abstract

SMSr/SAMD8 is an ER-resident ceramide phosphoethanolamine synthase with a critical role in controlling ER ceramides and suppressing ceramide-induced apoptosis in cultured cells. SMSr-mediated ceramide homeostasis relies on the enzyme’s catalytic activity as well as on its *N*-terminal sterile α-motif or SAM domain. Here we report that SMSr-SAM is structurally and functionally related to the SAM domain of diacylglycerol kinase DGKδ, a central regulator of lipid signaling at the plasma membrane. Native gel electrophoresis indicates that both SAM domains form homotypic oligomers. Chemical crosslinking studies show that SMSr self-associates into ER-resident trimers and hexamers that resemble the helical oligomers formed by DGKδ-SAM. Residues critical for DGKδ-SAM oligomerization are conserved in SMSr-SAM and their substitution causes a dissociation of SMSr oligomers as well as a partial redistribution of the enzyme to the Golgi. Conversely, treatment of cells with curcumin, a drug disrupting ceramide and Ca^2+^ homeostasis in the ER, stabilizes SMSr oligomers and promotes retention of the enzyme in the ER. Our data provide first demonstration of a multi-pass membrane protein that undergoes homotypic oligomerization via its SAM domain and indicate that SAM-mediated self-assembly of SMSr is required for efficient retention of the enzyme in the ER.

Many proteins have a modular organization in which distinct protein domains provide different functionalities. Sterile α-motif or SAM domains are widespread ~70 residue-long protein modules with essential roles in a broad variety of biological processes that include cell signaling, calcium homeostasis, synaptic scaffolding, transcriptional repression and translational control^1^,^2^,^3^. Although they adopt a similar fold, SAM domains are unusually versatile in their functional properties. SAM domains can self-assemble into polymers, form heterotypic complexes with SAM domains of other proteins or bind other protein interaction modules. For instance, the SAM domain of the transcriptional activator ETS-1 provides a docking site for the ERK-2 MAP kinase to facilitate its phosphorylation^4^. The SAM domain of the transcriptional repressor TEL, on the other hand, functions as a self-association motif that controls gene expression through extensive polymerization^5^. Isolated TEL-SAM domains form a head-to-tail polymer with six SAM monomers per turn. The SAM domain of diacylglycerol kinase-δ (DGKδ) forms a polymer with an architecture similar to that of TEL-SAM^6^,^7^. In unstimulated cells, DGKδ undergoes SAM-mediated self-assembly into cytosolic oligomers, which prevents its translocation to the plasma membrane. Stimulation of cells with epidermal growth factor results in oligomer disassembly and recruitment of DGKδ to the plasma membrane where the enzyme controls lipid signaling by altering the balance between the second messengers diacylglycerol (DAG) and phosphatidic acid^6^,^8^.

Besides their role in building homo- and heteromeric protein complexes, SAM domains have also been reported to bind RNA and lipids. Smaug in Drosophila and Vts1p in budding yeast control mRNA translation by binding to stem-loop structures in their target mRNAs via a cluster of positively charged residues in their SAM domains^9^,^10^. The SAM domain of p73, a homolog of the tumor suppressor p53, binds to membrane phospholipids^11^ whereas p63, another p53 homolog, shows interactions with the ganglioside GM1^12^. Because of their unique ability to recognize a large variety of binding partners, SAM domains cannot be easily categorized. This makes it necessary to analyze each SAM domain individually.

The mammalian proteome harbors two known multi-pass membrane proteins that contain a SAM domain, namely sphingomyelin synthase SMS1 and the SMS-related protein SMSr, also known as sterile α-motif domain-containing protein SAMD8. SMS1 is a Golgi-resident enzyme responsible for bulk production of sphingomyelin (SM), a major structural component of mammalian cell membranes and one of the end products of sphingolipid biosynthesis^13^,^14^. SMS1 catalyzes the transfer of phosphocholine from phosphatidylcholine onto ceramide in the Golgi lumen, a reaction yielding SM and DAG^15^,^16^. Thus, SMS1 occupies a key position in balancing the cellular levels of pro-apoptotic factor ceramide and mitogenic factor DAG. SMS1 depletion enhances ceramide production and apoptotic cell death after photodamage^17^, while its contribution to the formation of plasma membrane-associated SM is critical for Fas-clustering and Fas-mediated apoptosis^18^. SMS1 deficiency in mice causes moderate neonatal lethality as well as loss of fat tissue mass associated with impaired insulin secretion^19^,^20^. However, the functional relevance of the enzyme’s *N*-terminal SAM domain is not known.

Unlike SMS1, SMSr does not function as SM synthase but produces small quantities of the SM analogue ceramide phoshoethanolamine (CPE) in the lumen of the ER^21^,^22^. SMSr is the best-conserved member of the SMS enzyme family with homologs present in organisms such as *Drosophila*, which does not synthesize SM^23^. While the physiological relevance of SMSr-mediated CPE production in mammals remains to be established^22^,^24^, acute disruption of SMSr catalytic activity in cultured mammalian cells causes a substantial rise in ER ceramides and their mislocalization to mitochondria, triggering a mitochondrial pathway of apoptosis^21^,^25^. Interestingly, we found that SMSr-catalyzed CPE production, although required, is not sufficient to suppress ceramide-induced cell death and that SMSr-mediated ceramide homeostasis is critically dependent on the *N*-terminal SAM domain of the enzyme. Based on these results, we postulated a primary role of SMSr in monitoring ER ceramide levels to prevent untimely cell death during sphingolipid biosynthesis^25^.

To further dissect the mechanism by which SMSr controls ceramide levels in the ER, we here focused on the function of the enzyme’s *N*-terminal SAM domain. Our data disclose a striking structural and functional similarity between the SAM domains of SMSr and DGKδ. We show that SMSr-SAM drives the formation of homotypic SMSr trimers and hexamers, which resemble the helical oligomers formed by DGKδ-SAM. Moreover, we provide evidence that SAM-mediated self-assembly of SMSr is a critical determinant of the enzyme’s subcellular distribution, hence analogous to the role of SAM in DGKδ.

## Results

### SMSr-SAM lacks affinity for lipids

**Figure 1:**
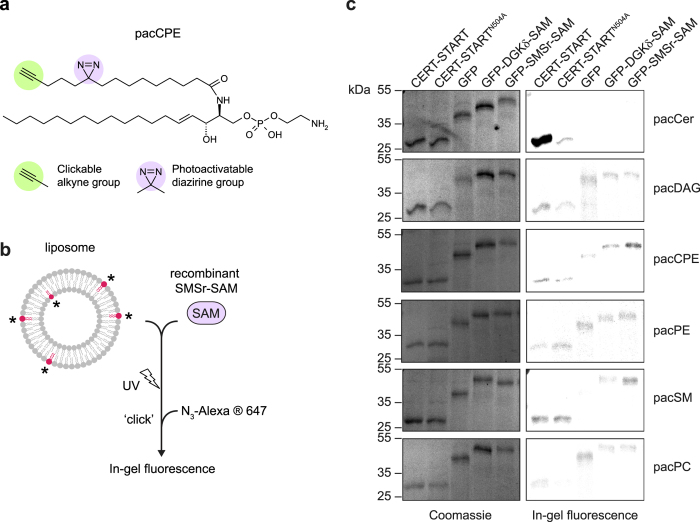
The SAM domain of SMSr lacks lipid-binding activity. (**a**) Photoactivatable and clickable analogue of ceramide phosphoethanolamine, pacCPE. (**b**) Schematic outline of lipid photoaffinity labeling assay. Recombinantly produced SAM domains are incubated with liposomes containing bifunctional lipid analogues and subjected to UV irradiation. Click chemistry is used to label the alkyne group in the bifunctional lipid with N_3_-AlexaFluor647, allowing visualization of UV-crosslinked protein–lipid complexes by in-gel fluorescence. (**c**) Recombinant GFP-fusions of the SAM domains of SMSr and diacylglycerol kinase DGKδ were subjected to lipid photoaffinity labeling using bifunctional analogues of ceramide (pacCer), diacylglycerol (pacDAG), CPE (pacCPE), phosphoethanolamine (pacPE), sphingomyelin (pacSM) and phosphatidylcholine (pacPC), processed for SDS-PAGE and analyzed by in-gel fluorescence (right) then stained with Coomassie (left). The ceramide-binding domain of ceramide transfer protein CERT (CERT-START) and a corresponding ceramide-binding mutant (CERT-START^N504A^) served as controls.

We previously demonstrated that SMSr is a CPE synthase that requires its *N*-terminal SAM domain to control ceramide levels in the ER^25^. As SAM domains have been reported to bind lipids^11^,^26^,^27^ including sphingolipids^12^, we performed photo-affinity labeling experiments with isolated SMSr-SAM produced in *E. coli* using bifunctional lipid analogues that contain a photo-activatable diazirine and a clickable alkyne group (Fig. 1a,b)^28^. Proteins in direct contact with a bifunctional lipid can be crosslinked by UV irradiation of the diazirine group. Next, click chemistry is used to label the alkyne group with a fluorescent reporter, allowing visualization of the crosslinked protein-lipid complex by in-gel fluorescence. As shown in Fig. 1c, the recombinant START domain of the ceramide transfer protein CERT could be specifically crosslinked with bifunctional ceramide. In contrast, recombinant SMSr-SAM was devoid of any specific lipid binding affinity when subjected to photo-crosslinking with bifunctional analogues of ceramide (Cer), CPE, SM, DAG, phosphatidylethanolamine (PE) or phosphatidylcholine (PC). This led us to explore alternative functions of SMSr-SAM than lipid binding.

### SMSr-SAM is structurally and functionally related to DGKδ-SAM

**Figure 2:**
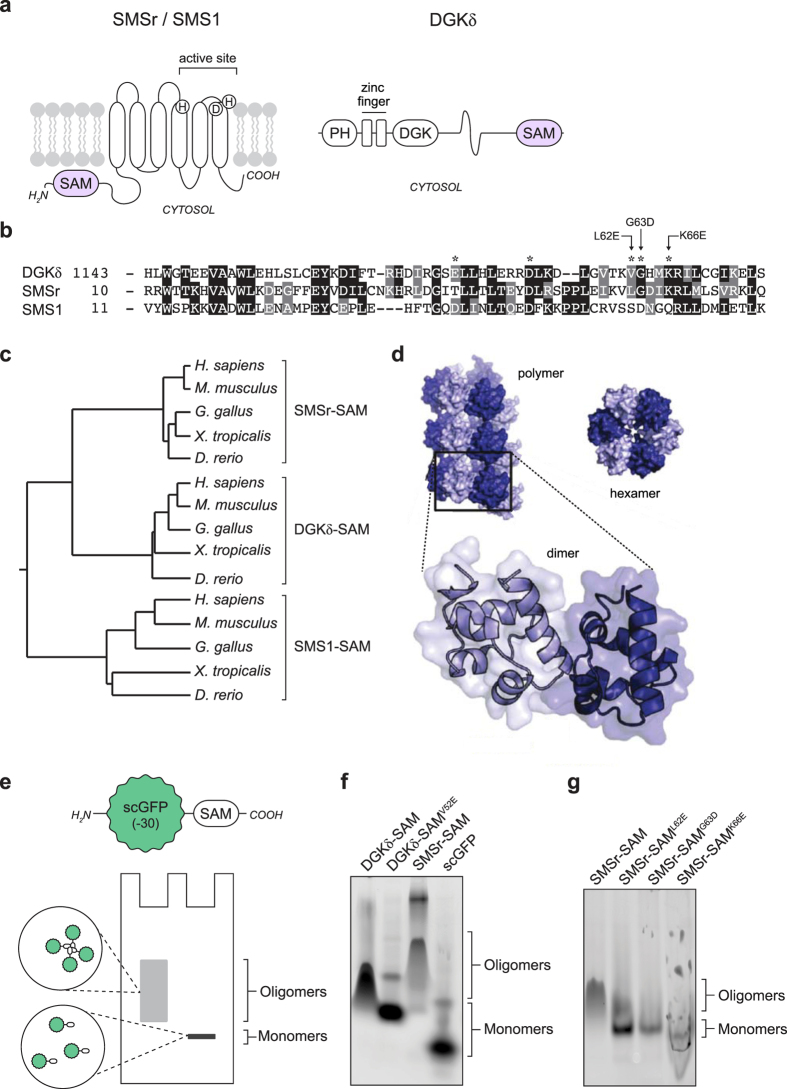
SMSr-SAM forms homo-oligomers *in vitro.* (**a**) Domain representations of SMSr, SMS1 and diacylglycerol kinase DGKδ. SMSr and SMS1 have six predicted membrane spans, an active site facing the exoplasmic leaflet and an *N*-terminal SAM domain exposed to the cytosol. DGKδ is a cytosolic protein with an *N*-terminal pleckstrin homology (PH) domain and a *C*-terminal SAM domain. (**b**) Sequence alignment of human DGKδ-SAM (Uniprot: Q16760), SMSr-SAM (Q96LT4) and SMS1-SAM (Q86VZ5). Note that residues critical for homo-oligomerization of DGKδ-SAM (marked with an asterisk) are largely conserved in SMSr-SAM but not in SMS1-SAM. (**c**) Phylogenetic analysis of SAM domains of vertebrate homologs of DGKδ, SMSr and SMS1. Sequences were aligned using the Clustal Omega multiple sequence alignment tool^55^ and the phylogenetic tree was drawn using ClustalW2 Phylogeny. Database accession numbers used are provided in Supplementary Table 2. (**d**) Space-filling model of the DGKδ-SAM polymer (adapted with permission from Knight M.J. *et al*. Biochemistry 2010, 49:9667-9676, copyright 2010, American Chemical Society). (**e**) Schematic outline of a native gel assay to screen SAM domains for their ability to form homo-oligomers. Lysates of bacteria expressing SAM domains fused to super-negatively charged GFP (scGFP) are run on a native gel and analyzed by in-gel fluorescence. The strong negative charge of scGFP helps solubilize SAM domains and drive their migration to the anode. (**f**) Bacterial lysates containing scGFP fusions of DGKδ-SAM, oligomerization mutant DGKδ-SAM^V52E^ or SMSr-SAM were run on a native gel and analyzed by in-gel fluorescence. Note that SMSr-SAM, analogous to DGKδ-SAM but contrary to DGKδ-SAM^V52E^, displays a retarded migration indicative of protein oligomers. (**g**) Bacterial lysates containing scGFP fusions of SMSr-SAM, SMSr-SAM^L62E^, SMSr-SAM^G63D^ or SMSr-SAM^K66E^ were run on a native gel and analyzed by in-gel fluorescence. Note that all SMSr-SAM point mutants display a dramatic shift in gel migration compared to wild-type SMSr-SAM, indicative of a perturbation in protein oligomerization.

A BLAST search with SMSr-SAM as query yielded the SAM domain of DAG kinase DGKδ as a hit with the lowest Expect (E) value (i.e. 3E-05) (Suppl. Table 1; Fig. 2a,b). In addition, phylogenetic analysis revealed that SMSr-SAM is more closely related to DGKδ-SAM than to the SAM domain of SMS1 (Fig. 2c). Isolated DGKδ-SAM has been shown to self-assemble into helical oligomers through a head-to-tail interaction, with six SAM monomers per turn^7^ (Fig. 2d). SAM-mediated oligomerization of DGKδ controls its function as a key regulator of lipid signaling by sequestering the enzyme in an inactive cellular location^6^. Moreover, several key residues involved in DGKδ-SAM homo-oligomerization are conserved in SMSr-SAM from human to zebrafish, but do not occur in SMS1-SAM (Fig. 2b; residues marked by asterisks). This suggested that SMSr-SAM and DGKδ-SAM share a similar function.

To investigate whether SMSr-SAM, like DGKδ-SAM, is able to self-associate into oligomers, we used a method originally developed by Knight *et al*.^29^. To this end, SMSr-SAM and DGKδ-SAM were expressed in bacteria as fusions to a super-negatively charged GFP (scGFP) and run on a native gel (Fig. 2e). The negative charge on GFP minimizes the chance of GFP-mediated self-assembly and ensures that all proteins migrate to the cathode while the green fluorescence can be used to monitor the migration of the fusion proteins in the native gel. As shown in Fig. 2f, polymeric scGFP-DGKδ-SAM displayed a major shift in migration relative to its monomeric control, scGFP-DGKδ-SAM^V52E^. In comparison to scGFP-DGKδ-SAM, scGFP-SMSr-SAM migrated even slower through the gel while a significant portion of the protein remained in the gel well (data not shown). These results are consistent with those reported by Knight *et al*.^29^ and suggested that scGFP-SMSr-SAM forms stable oligomers. To verify that oligomerization is mediated by the SAM domain, we mutated each of three conserved residues that were previously shown to be critical for homo-oligomerization of DGKδ-SAM, yielding SMSr-SAM^L62E^, SMSr-SAM^G63D^ and SMSr-SAM^K66E^ (Fig. 2b). All three mutants migrated faster through the gel than wild-type SMSr-SAM (Fig. 2g), indicative of a defect in homo-oligomerization. From this we conclude that SMSr-SAM is able to form homo-oligomers.

### SMSr forms SAM-dependent oligomers in the ER

**Figure 3:**
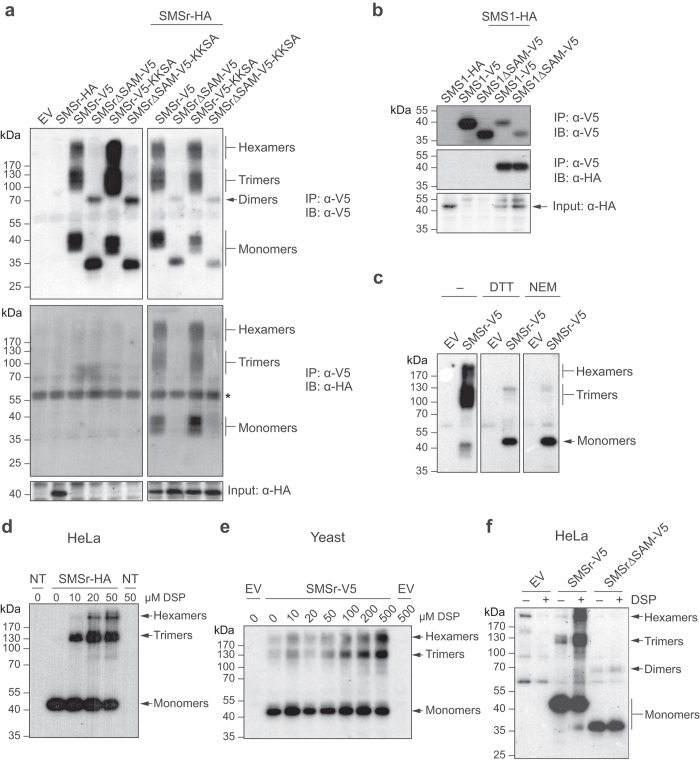
SMSr forms SAM-dependent homo-oligomers in the ER. (**a**) Detergent extracts of HeLa cells co-transfected with HA-tagged SMSr and V5/His6-tagged SMSr, SMSr∆SAM, SMSr-KKSA or SMSr∆SAM-KKSA were subjected to immunoprecipitation analysis using an anti-V5 antibody. Immunoprecipitates (IP) and total extracts (input) were immunoblotted (IB) using anti-V5 and anti-HA antibodies. An asterisk denotes immunoreactivity with IgG heavy chain. (**b**) Detergent extracts of HeLa cells co-transfected with HA-tagged SMS1 and V5/His6-tagged SMS1 or SMS1∆SAM were subjected to immunoprecipitation analysis using an anti-V5 antibody. Immunoprecipitates (IP) and total extracts (input) were immunoblotted (IB) using anti-V5 and anti-HA antibodies, as in (**a**). (**c**) HeLa cells transfected with empty vector (EV) or V5/His6-tagged SMSr were solubilized using detergent in the presence or absence of 10 mM *N*-ethyl maleimide (NEM) and subjected to Ni^2+^ -NTA affinity chromatography. Ni^2+^-NTA eluates were incubated in the presence or absence of 100 mM DTT, and immunoblotted (IB) using an anti-V5 antibody, as in (**a**). (**d**) HeLa cells transfected with HA-tagged SMSr were treated with chemical crosslinker DSP (0–50 μM, 15 min, RT), solubilized by detergent in the presence of 10 mM NEM and subjected to immunoprecipitation analysis using an anti-HA antibody. Immunoprecipitates were immunoblotted using an anti-HA antibody. (**e**) Yeast cells transfected with empty vector (EV) or V5/His6-tagged SMSr were treated with DSP as in (**d**), lysed and subjected to immunoblot analysis using an anti-V5 antibody. (**f**) HeLa cells transfected with empty vector (EV), V5/His6-tagged SMSr or SMSr∆SAM were incubated in the presence or absence of DSP (50 μM, 15 min, RT), solubilized with detergent in the presence of 10 mM NEM and subjected to Ni^2+^-NTA affinity chromatography. Ni^2+^-NTA eluates were immunoblotted using an anti-V5 antibody. Uncropped images of blots are provided in Supplementary Fig. S1.

To investigate whether SMSr forms homo-oligomers in cells, we performed co-immunoprecipitation (IP) experiments on HeLa cells co-expressing V5- and HA-tagged versions of the enzyme. Immunoblot analysis of anti-V5 immunoprecipitates revealed, besides monomeric SMSr, also higher-order enzyme complexes that were resistant to SDS under non-reducing conditions (Fig. 3a). The migration profiles of the SDS-resistant enzyme complexes and their cross-reactivity with both anti-V5 and anti-HA antibodies suggested that they correspond to SMSr trimers and hexamers (see also below). When immunoprecipitations were performed on HeLa cells co-expressing full-length SMSr-HA and a truncated version of SMSr-V5 lacking the *N*-terminal SAM-domain, the anti-V5 immunoprecipitates were devoid of SMSr-HA and only contained SMSrΔSAM monomers and dimers (Fig. 3a). Thus, the ability of SMSr to form trimers and hexamers appeared critically dependent on its SAM domain. We previously reported that removal of its SAM domain causes SMSr to redistribute from the ER to the Golgi^25^ (see also below). To address the possibility that immunoprecipitation of SMSrΔSAM-V5 fails to bring down SMSr-HA because both proteins reside in different organelles, SMSrΔSAM-V5 was retained in the ER by adding a KKXX ER-retrieval motif to its *C*-terminus. As shown in Fig. 3a, anti-SMSrΔSAM-V5-KKSA immunoprecipitates were still devoid of SMSr-HA. In contrast, co-immunoprecipitation analysis on cells co-expressing full-length and SAM-truncated versions of SMS1 revealed that this enzyme self-associates independently of its SAM domain (Fig. 3b). Thus, consistent with its ability to self-associate *in vitro*, SMSr-SAM appears to drive homo-oligomerization of SMSr in the ER.

We noticed that under non-reducing conditions, SMSr monomers, trimers and hexamers all migrate as discrete smears in the gel. Treatment of immunoprecipitates with DTT prior to gel electrophoresis dissolved most of the trimers and hexamers, and caused the monomer to migrate as a single protein band (Fig. 3c), indicating that the electrophoretic mobility of SMSr is strongly influenced by the presence of both intermolecular and intramolecular disulfide bonds. To investigate whether these disulfide bonds form before or after cell lysis, cells were lysed in the presence of *N*-ethylmaleimide (NEM), a membrane permeable agent that reacts with free thiol groups. NEM treatment recapitulated the effect of DTT addition (Fig. 3c), indicating that the intermolecular and intramolecular disulfide bonds in SMSr form after cell lysis, presumably due to the oxidative milieu provided by the lysis buffer. Henceforth, NEM was included in the lysis buffer in all subsequent experiments. To verify that SMSr also forms trimers and hexamers in intact cells, HeLa cells expressing SMSr-HA were treated with the membrane permeable and amine-reactive crosslinker DSP prior to immunopreciptation analysis. As shown in Fig. 3d, DSP treatment led to the appearance of SMSr trimers and hexamers in anti-HA immunoprecipitates. These trimers and hexamers became more abundant at the expense of monomers when increasing amounts of crosslinker were used. SMSr trimers and hexamers could also be recovered from immunoprecipitates of DSP-treated and SMSr-V5-expressing *Saccharomyces cerevisiae*, an organism lacking endogenous SMSr homologs. Interestingly, some trimers and hexamers were also observed when crosslinker was omitted (Fig. 3e). In contrast, immunoprecipitates prepared from DSP-treated HeLa cells expressing SMSrΔSAM-V5 contained monomers and dimers but were devoid of any trimers or hexamers (Fig. 3f). From this we conclude that SMSr-SAM mediates self-assembly of SMSr into trimers and hexamers in the ER. SMSr lacking its *N*-terminal SAM-domain, on the other hand, appears to retain the ability to form dimers.

### Isolation and functional analysis of oligomerization-defective SMSr mutants

**Figure 4:**
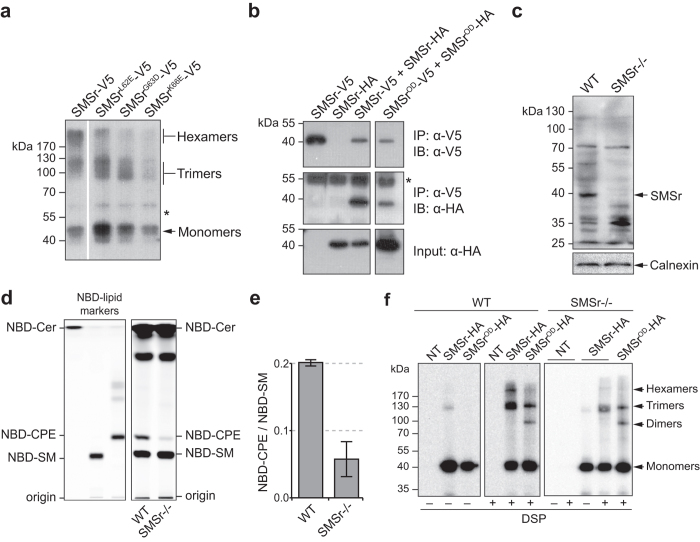
Functional analysis of oligomerization-defective SMSr mutants. (**a**) Detergent extracts of HeLa cells transfected with V5-tagged SMSr, SMSr^L62E^, SMSr^G63D^ and SMSr^K66E^ were subjected to immunoprecipitation and immunoblotting using an anti-V5 antibody. An asterisk denotes immunoreactivity with IgG heavy chain. (**b**) HeLa cells co-transfected with V5/His6-tagged and HA-tagged SMSr or SMSr^L62E/G63D/K66E^ (SMSr^OD^) were solubilized with detergent in the presence of 10 mM NEM and subjected to immunoprecipitation analysis using an anti-V5 antibody. Immunoprecipitates (IP) and total extracts (input) were immunoblotted (IB) using anti-V5 and anti-HA antibodies. An asterisk denotes immunoreactivity with IgG heavy chain. (**c**) Total membranes of wild-type (WT) and SMSr knockout (SMSr^−/−^) HeLa cells were subjected to immunoblot analysis using anti-SMSr and anti-calnexin antibodies. (**d**) TLC analysis of reaction products formed when lysates of WT and SMSr^−/−^ HeLa cells were incubated with C_6_-NBD-ceramide (NBD-Cer). Note that lysates of SMSr^−/−^ cells have a substantially reduced capacity to synthesize NBD-CPE. (**e**) Ratio of quantified NBD-Cer-derived reaction products from (**c**). Data shown are the means of two independent experiments. (**f**) WT and SMSr^−/−^ HeLa cells transfected with HA-tagged SMSr or SMSr^OD^ were incubated in the presence or absence of DSP (50 μM, 15 min, RT), solubilized with detergent in the presence of 10 mM NEM and subjected to immunoprecipitation analysis using an anti-HA antibody. Immunoprecipitates were immunoblotted using an anti-HA antibody. NT, non-transfected. Uncropped images of blots are provided in Supplementary Fig. S2.

To study the functional relevance of SMSr oligomerization in HeLa cells, we first generated an oligomerization-defective SMSr mutant. IP analysis of V5-tagged SMSr mutants carrying one of three single residue substitutions that blocked SMSr-SAM oligomerization *in vitro* (Fig. 2g) revealed that each of these reduced the ability of the enzyme to self-associate into trimers and hexamers (Fig. 4a). When all three single residue substitutions were combined, we observed a substantial reduction in the self-associating properties of SMSr as judged by co-IP analysis (Fig. 4b). We named this oligomerization-defective triple mutant SMSr^OD^. To allow a detailed functional analysis of SMSr^OD^ without interference from any endogenous pool of oligomerization-competent SMSr, we created HeLa SMSr knockout (SMSr^−/−^) cells using CRISPR/Cas9 technology. Contrary to wild-type cells, SMSr^−/−^ cells lack a 39-kDa protein that cross-reacts with a well-characterized anti-SMSr antibody^22^ (Fig. 4c). Moreover, SMSr^−/−^ cells were virtually devoid of CPE synthase activity (Fig. 4d,e). This is consistent with previous RNAi experiments indicating that SMSr is the principal CPE synthase in HeLa cells^21^. The residual CPE synthase activity detected in SMSr^−/−^ cells is likely due to SMS2, which serves as a bifunctional enzyme with both SM and CPE synthase activity^30^. Unlike HeLa cells treated with SMSr-targeting siRNAs, SMSr^−/−^ cells lack any signs of apoptosis (our unpublished data). Whether this is due to a compensatory mechanism that overcomes a deregulation of ER ceramides over time remains to be established. As shown in Fig. 4f, SMSr^OD^ displayed a reduced ability to self-associate into hexamers and trimers following its immunoprecipitation from either DSP-treated wild-type or SMSr^−/−^ cells. Besides a marked decrease in the recovery of hexamers and trimmers, DSP-crosslinking of SMSr^OD^ led to appearance of dimers (Fig. 4f). Dimers were also observed in crosslinking experiments with the SAM-domain truncation mutant but not with the wild-type enzyme (Fig. 3f). Together, these results provide complementary evidence that SAM-mediated self-assembly is the key mechanism by which SMSr forms trimers and hexamers in the ER.

### SMSr oligomerization is dispensable for catalytic activity and vice versa

**Figure 5:**
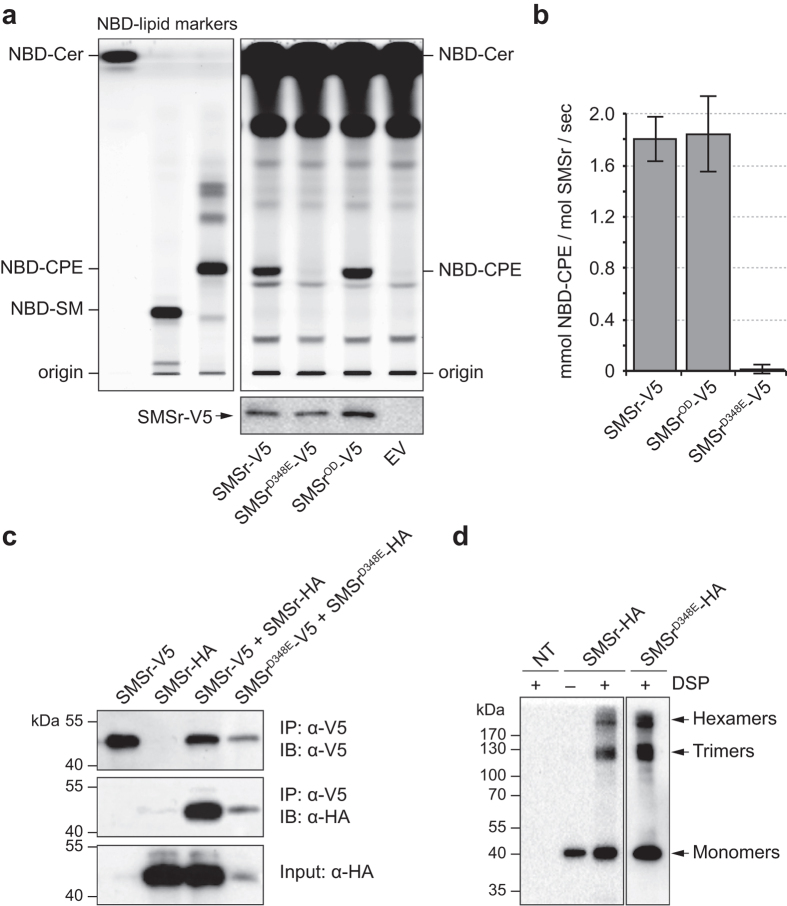
SMSr oligomerization is dispensable for catalytic activity and vice versa. (**a**) TLC analysis of reaction products formed when lysates of yeast cells expressing V5/His6-tagged SMSr, enzyme-dead SMSr^D348E^ or SMSr^OD^ were incubated with NBD-Cer (top). SMSr expression was verified by immunoblotting using an anti-V5 antibody (bottom). EV, empty vector. (**b**) Specific activities of V5/His6-tagged SMSr, SMSr^OD^ and enzyme-dead SMSr^D348E^ in yeast were determined by quantitative immunoblotting and TLC analysis of reaction products formed when lysates were incubated with NBD-Cer and expressed as mmol NBD-CPE formed per mol SMSr per second. Data shown are the mean ± S.D. of three independent experiments performed in duplicate. (**c**) SMSr^−/−^ HeLa cells co-transfected with V5/His6-tagged and HA-tagged SMSr or SMSr^D348E^ were solubilized with detergent in the presence of 10 mM NEM and subjected to immunoprecipitation analysis using an anti-V5 antibody. Immunoprecipitates (IP) and total extracts (input) were immunoblotted (IB) using anti-V5 and anti-HA antibodies. (**d**) SMSr^−/−^ HeLa cells were transfected with HA-tagged SMSr or SMSr^D348E^ and incubated in the presence or absence of DSP (50 μM, 15 min, RT), solubilized with detergent in the presence of 10 mM NEM and subjected to immunoprecipation analysis using an anti-HA antibody. Immunoprecipitates were immunoblotted using an anti-HA antibody. NT, non-transfected. Uncropped images of blots are provided in Supplementary Fig. S3.

To address whether an impaired capacity to form homo-oligomers influences the catalytic activity of SMSr, we expressed V5-tagged SMSr, SMSr^OD^ and a catalytically dead SMSr^D348E^ mutant in *Saccharomyces cerevisiae*, an organism lacking endogenous CPE synthase activity. Next, catalytic activities of the heterologously expressed enzymes were determined in cell lysates using a recently established quantitative CPE synthase activity assay^31^. Whereas the D348E mutation completely abolished SMSr-mediated CPE production, there was no significant difference between the catalytic activities of SMSr and SMSr^OD^, indicating that the oligomeric state of the enzyme does not critically influence its catalytic capacity (Fig. 5a,b). We then addressed whether disrupting catalytic activity influences the ability of SMSr to form homo-oligomers. Although introduction of the D348E mutation appeared to render SMSr less stable when expressed in HeLa cells, co-IP analysis of SMSr^−/−^ cells co-expressing V5- and HA-tagged SMSr^D348E^ showed that catalytic activity per se is not a prerequisite for SMSr self-assembly (Fig. 5c). Moreover, DSP crosslinking experiments revealed that SMSr^D348E^ displays the same capacity to self-assemble into hexamers and trimers as the wild-type enzyme (Fig. 5d). Collectively, these results indicate that the ability of SMSr to form homo-oligomers is dispensable for its catalytic activity and vice versa.

### Curcumin promotes SMSr oligomerization

**Figure 6:**
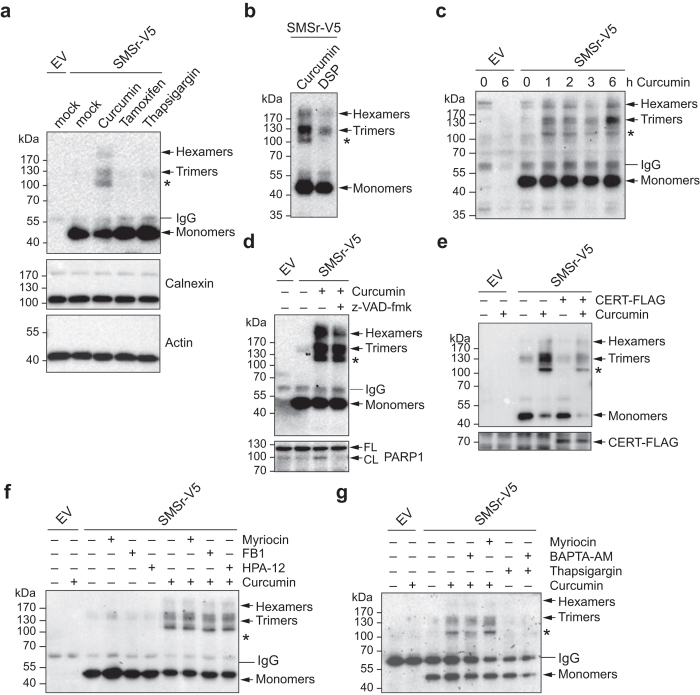
Curcumin promotes SMSr oligomerization independently of fluctuations in ER ceramide or Ca^2+^ levels. (**a**) HeLa cells transfected with empty vector (EV) or V5/His6-tagged SMSr were treated with vehicle (mock), curcumin (50 μM), tamoxifen (20 μM) or thapsigargin (2 μM) for 6 h, solubilized with detergent in the presence of 10 mM NEM and subjected to immunoprecipation analysis using anti-V5 antibody. Immunoprecipitates (top) or total extracts (middle, bottom) were immunoblotted using anti-V5 (top), anti-calnexin (middle) and anti-actin antibodies (bottom). (**b**) HeLa cells transfected with V5/His6-tagged SMSr were treated with curcumin (50 μM, 6 h) or DSP (50 μM, 15 min) and subjected to immunoprecipation and immunoblot analysis using anti-V5 antibody. (**c**) HeLa cells transfected with empty vector (EV) or V5/His6-tagged SMSr were treated with 50 μM curcumin for the indicated time and analyzed as in (**b**). (**d**) HeLa cells transfected with empty vector (EV) or V5/His6-tagged SMSr were treated with 50 μM curcumin in the presence or absence of 20 μM of pan-caspase inhibitor zVAD-fmk for 6 h and then subjected to immunoprecipation analysis using anti-V5 antibody. Immunoprecipitates and total extracts were immunoblotted using anti-V5 (top) and anti-PARP1 antibodies (bottom), respectively. FL, full length; CL, cleaved. (**e**) HeLa cells co-transfected with V5/His6-tagged SMSr and FLAG-tagged CERT or empty vector (EV) were treated with 50 μM curcumin for 1 h, solubilized with detergent in the presence of 10 mM NEM and subjected to Ni^2+^-NTA affinity chromatography. Ni^2+^-NTA eluates and total extracts were immunoblotted using anti-V5 (top) and anti-FLAG antibodies (bottom), respectively. (**f**) HeLa cells transfected with empty vector (EV) or V5/His6-tagged SMSr were pre-incubated with 1 μM myriocin, 20 μM Fuminosin B1 or 2.5 μM HPA-12 for 5 h and then treated with 50 μM curcumin for 1 h, where indicated. Cells were processed as in (**e**). (**g**) HeLa cells transfected with empty vector (EV) or V5/His6-tagged SMSr were pre-incubated with 100 nM myriocin (1 h) or 50 μM BAPTA-AM (5 min) and then treated with curcumin (50 μM) or thapsigargin (20 μM) for 1 h. Cells were processed as in (**e**). A heterologous SMSr-protein complex of ~100 kDa formed in curcumin-treated cells is marked by an asterisk. Uncropped images of blots are provided in Supplementary Fig. S4.

As SMSr is a critical regulator of ER ceramides^21^,^25^ and because ceramides can trigger ER stress by disrupting Ca^2+^ homeostasis^32^, we wondered whether an acute imbalance in ER ceramide or Ca^2+^ levels would influence the oligomeric state of the enzyme. Several drugs have been reported to acutely alter ceramide and/or Ca^2+^ levels in the ER. For instance, curcumin stimulates *de novo* ceramide biosynthesis, presumably by directly activating ceramide synthases in the ER^33^,^34^,^35^. Tamoxifen has been reported to increase ceramide levels by blocking glucosylceramide biosynthesis^36^,^37^ whereas thapsigargin is known to release Ca^2+^ from the ER lumen by inhibiting the sarco/endoplasmic reticulum Ca^2+^-ATPase, SERCA^38^. As shown in Fig. 6a, treatment of SMSr-V5-expressing HeLa cells with curcumin led to the appearance of higher-order enzyme complexes in anti-V5 immunoprecipitates that were resistant to SDS-PAGE under reducing conditions. No such complexes were observed in immunoprecipitates from control cells or from cells treated with tamoxifen or thapsigargin. Curcumin treatment did not trigger formation of higher-order complexes of calnexin, an abundant membrane-anchored chaperone of the ER. The curcumin-induced SMSr complexes co-migrated, at least in part, with DSP-crosslinked SMSr trimers and hexamers (Fig. 6b), indicating that curcumin promotes the formation of SDS-resistant SMSr homo-oligomers. A distinct SMSr-containing complex of approximately 110 kDa was consistently and exclusively found in curcumin-treated cells (marked with an asterisk in Fig. 6a,b). As this curcumin-induced complex did not match the migration profiles of SMSr dimers (~85 kDa), trimers (~125 kDa) or hexamers (~250 kDa), we anticipate that it represents a heterologous complex comprising SMSr and another, yet-to-be identified protein. Identification of this SMSr interaction partner is the subject of ongoing investigations.

### Curcumin-induced SMSr oligomerization is independent of fluctuations in ER ceramide or calcium levels

As curcumin has been reported to trigger mitochondrial apoptosis^33^,^35^,^39^, we first examined whether its ability to promote SMSr homo-oligomerization relies on activation of an apoptotic pathway. A time course experiment revealed that curcumin triggers SMSr oligomerization already within one hour (Fig. 6c). Moreover, treatment of cells with the pan-caspase inhibitor zVAD-fmk blocked curcumin-induced PARP cleavage but did not prevent formation of SDS-resistant SMSr oligomers (Fig. 6d). Together, these results indicate that curcumin promotes SMSr homo-oligomerization independently of its apoptogenic activity.

Since curcumin stimulates *de novo* ceramide biosynthesis, we next investigated whether formation of SDS-resistant SMSr oligomers in curcumin-treated cells is due to an accumulation of ceramides in the ER. We previously reported that over-expression of the ceramide transfer protein CERT rescues SMSr-depleted cells from ceramide-induced apoptosis by stimulating ceramide export from the ER^25^. As shown in Fig. 6e, CERT over-expression did not prevent formation of SDS-resistant SMSr oligomers in curcumin-treated cells. We then used various well-characterized drugs that block either *de novo* ceramide biosynthesis or ceramide export from the ER. As shown in Fig. 6f, long chain base synthase inhibitor myriocin or ceramide synthase inhibitor fumonisin B1 in each case had no impact on curcumin-induced SMSr oligomerization when added 5 h prior to curcumin treatment. Also, addition of HPA-12, a specific inhibitor of CERT-mediated ceramide transport^40^, did not lead to any obvious changes in the oligomeric state of SMSr in either control or curcumin-treated cells (Fig. 6f). From this we conclude that curcumin-induced oligomerization of SMSr occurs independently of fluctuations in ER ceramide levels.

Besides deregulating ceramide levels in the ER, curcumin promotes the release of Ca^2+^ from the ER lumen by inhibiting SERCA^41^. BAPTA-AM, a membrane permeable Ca^2+^ chelator, has been reported to block some of the downstream effects of curcumin^42^,^43^. However, pre-treatment of cells with BAPTA-AM did not prevent curcumin-induced SMSr oligomerization (Fig. 6g). Moreover, addition of thapsigargin, a specific inhibitor of SERCA, did not mimic the effect of curcumin on SMSr oligomerization. This indicates that curcumin-induced formation of SDS-resistant SMSr oligomers is unlikely mediated by a perturbed Ca^2+^ homeostasis in the ER. Curcumin has previously been reported to crosslink the cystic fibrosis transmembrane conductance receptor into SDS-resistant oligomers^44^ and to promote dimerization of ceramide synthases^34^. Thus, the appearance of SDS-resistant SMSr oligomers in cells treated with curcumin may be due to the crosslinking activity of this drug.

### SMSr oligomerization is critical for ER localization

**Figure 7:**
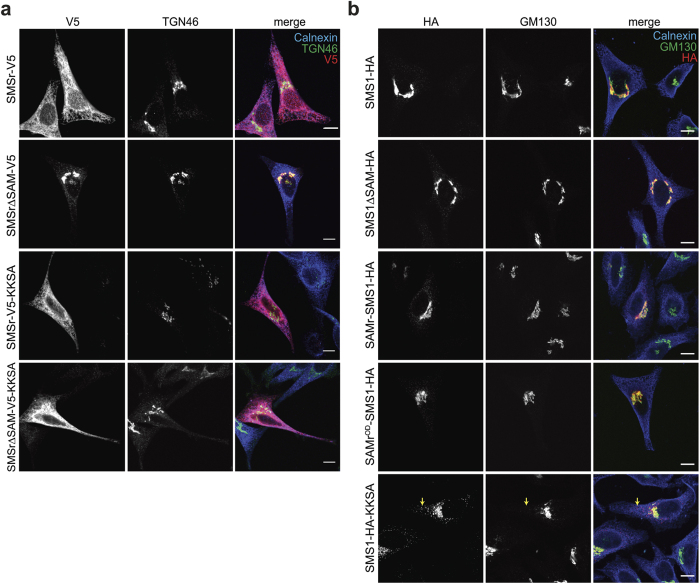
Effect of SAM removal or swapping on the subcellular distribution of SMSr and SMS1. (**a**) Confocal sections of HeLa cells transfected with V5/His6-tagged SMSr, SMSr∆SAM, SAMr-KKSA, or SMSr∆SAM-KKSA. Cells were immunostained using mouse anti-V5 (red), sheep anti-TGN46 (green) and rabbit anti-calnexin primary antibodies (blue) followed by Cy3-conjugated donkey anti-mouse, FITC-conjugated donkey anti-sheep and Cy5-conjugated donkey anti-rabbit secondary antibodies. Bar, 10 μm. (**b**) Confocal sections of HeLa cells transfected with HA-tagged SMS1, SMS1∆SAM, SAMr-SMS1, SAMr^OD^-SMS1 or SMS1-KKSA. Cells were immunostained using rabbit anti-HA (red), mouse anti- GM130 (green) and goat anti-calnexin primary antibodies (blue) followed by Cy3-conjugated donkey anti-rabbit, Cy2-conjugated donkey anti-mouse and Cy5-conjugated donkey anti-goat secondary antibodies. Bar, 10 μm.

The subcellular distribution of DGKδ is controlled by its SAM domain, which prevents translocation of the enzyme to the plasma membrane by mediating its self-assembly into cytosolic oligomers^6^. Removal of its *N*-terminal SAM domain caused SMSr to redistribute from the ER to the Golgi complex^21^ (Fig. 7a). In contrast, removal of the *N*-terminal SAM domain from SMS1 did not affect its Golgi residency (Fig. 7b). Swapping the entire *N*-terminal cytoplasmic tail of SMS1 against that of SMSr, on the other hand, caused the protein to be retained in the ER^21^. To investigate whether SAM-mediated homo-oligomerization of SMSr plays a role in retaining the enzyme in the ER, we created chimeric SMS1 proteins in which the SAM domain is swapped for SMSr-derived wild-type or oligomerization-defective SAM. We named these chimeric proteins SAMr-SMS1 and SAMr^OD^-SMS1, respectively. As shown in Fig. 7b, both SAMr-SMS1 and SAMr^OD^-SMS1 exclusively localized to the Golgi complex. Collectively, these results indicated that SMSr-SAM does not mediate ER retention or, alternatively, that such an activity is obscured by the presence of an ER export signal in the *N*-terminal cytoplasmic tail of SMS1 located downstream of its SAM domain. The latter scenario is supported by our finding that addition of a *C*-terminal KKXX ER-retrieval sequence caused a redistribution of SMSrΔSAM from the Golgi to the ER, but failed to retain SMS1 in the ER (Fig. 7a,b).

**Figure 8:**
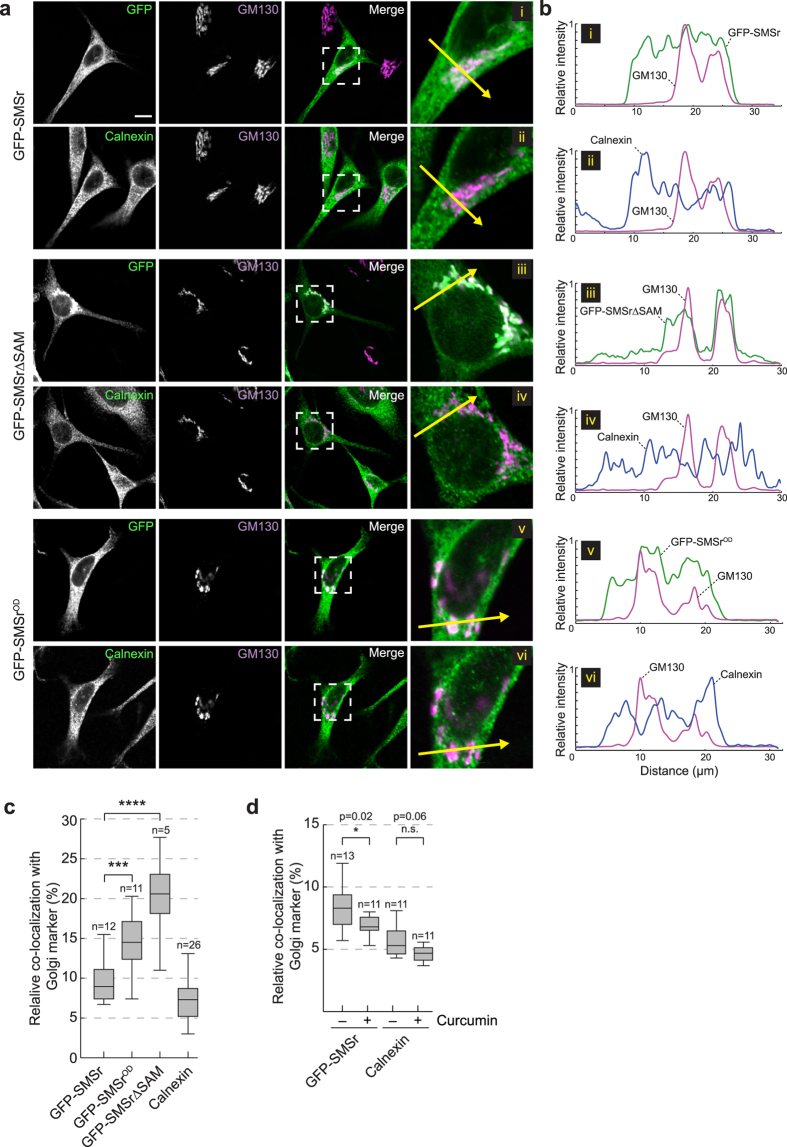
SMSr oligomerization is critical for ER localization. (**a**) Confocal sections of SMSr^−/−^ HeLa cells transfected with GFP-tagged SMSr, SMSr∆SAM or SMSr^OD^. Cells were immunostained using mouse anti-GM130 (magenta) and rabbit anti-calnexin primary antibodies (green) followed by Cy3-conjugated donkey anti-mouse and Cy5-conjugated donkey anti-rabbit secondary antibodies. Bar, 10 μm. (**b**) Intensity plots along the paths of the arrows in (**a**), showing overlap between GFP or anti-calnexin (green) and anti-GM130 (magenta) channels. (**c**) Manders’ correlation coefficients of fluorescence-intensity-based colocalization of GFP-tagged SMSr, SMSr^OD^ or SMSr∆SAM with the Golgi marker GM130 was calculated from confocal scanning of complete z-stacks of cells treated as in (**a**). (**d**) Manders’ correlation coefficients of fluorescence-intensity-based colocalization of GFP-tagged SMSr or calnexin with the Golgi marker GM130 was calculated from confocal scanning of complete z-stacks of control or curcumin-treated cells (50 μM, 1 h). For each boxplot in (**c**) and (**d**), the middle line denotes the median and the top and bottom of the box indicate the 75th and 25th percentile. The whiskers denote the maximum and minimum values. P-values of unpaired t-test: *P < 0.05, ***P < 0.001, ****P < 0.0001.

As alternative approach to investigate whether SAM-mediated oligomerization is part of the mechanism by which SMSr is retained in the ER, we next analyzed the subcellular distribution of GFP-tagged SMSr, SMSr∆SAM and SMSr^OD^ in HeLa SMSr^−/−^ cells using confocal fluorescence microscopy. As shown in Fig. 8a, GFP-SMSr primarily resides in the ER while a minor but significant pool of the enzyme is localized in the Golgi complex. Indeed, intensity plots along a line that crosssections the Golgi complex demonstrated partially overlapping intensity peaks for GFP-SMSr and the Golgi marker GM130; such overlap was not observed between intensity plots for GM130 and the ER marker calnexin (Fig. 8b). In contrast to GFP-SMSr and in line with our previous findings, the bulk of GFP-SMSr∆SAM localized to the Golgi complex with residual amounts remaining in the ER. GFP-SMSr^OD^, on the other hand, displayed a subcellular distribution intermediate to that of GFP-SMSr and GFP-SMSr∆SAM, with sizeable portions of the enzyme present in both ER and Golgi (Fig. 8a,b). This became even more apparent when we quantified the relative portion of GFP-tagged enzyme that co-localized with the Golgi marker GM130 using Manders’ co-efficient^45^ (Fig. 8c). Thus, mutations that perturb SMSr homo-oligomerization promote a redistribution of the enzyme from the ER to the Golgi complex. Interestingly, we found that treatment of cells with curcumin caused the opposite trend by promoting a redistribution of the enzyme from the Golgi to the ER (Fig. 8d). Together, these results indicate that SAM-mediated homo-oligomerization of SMSr serves a critical role in retaining the enzyme in the ER.

## Discussion

Previous work revealed that SMSr, a CPE synthase with potential dual activity as ceramide sensor, requires its *N*-terminal SAM domain to control ER ceramide levels and suppress ceramide-induced mitochondrial apoptosis. In the present study, we uncovered a striking structural and functional similarity between SMSr-SAM and the SAM domain of the diacylglycerol kinase DGKδ. We show that SMSr-SAM, analogous to DGKδ-SAM, drives self-assembly of the enzyme into oligomers and that SAM-mediated oligomerization is a critical determinant of the enzyme’s subcellular localization. Thus, our study provides the first example of a polytopic membrane protein that undergoes homotypic oligomerization via its SAM domain and indicates that self-association of SMSr is part of the mechanism by which the enzyme is retained in the ER.

Complementary lines of evidence indicate that the SAM domain of SMSr mediates self-assembly of the enzyme into oligomers. To begin with, SMSr-SAM is structurally and functionally related to the SAM domain of DGKδ, an enzyme that forms homotypic oligomers in the cytosol via its SAM domain to control its function as regulator of lipid signaling at the plasma membrane^8^. Residues critical for oligomerization of DGKδ-SAM are conserved in SMSr-SAM and substitution of these residues abolishes self-assembly of isolated SMSr-SAM *in vitro* and weakens the formation of SMSr homotypic oligomers *in vivo*. Moreover, chemical crosslinking studies indicate that SMSr self-assembles into trimers and hexamers, thus resembling the helical oligomers formed by DGKδ-SAM, which comprise six monomers per turn^6^. SMSr expressed in budding yeast, an organism that lacks structural homologs of SMS enzymes, forms higher-order complexes that co-migrate with SMSr trimers and hexamers assembled in human cells, in line with the idea that SMSr oligomerization involves self-assembly of the enzyme through its SAM domain and does not rely on any auxiliary protein.

SAM-mediated oligomerization of DGKδ prevents translocation of the enzyme to the plasma membrane, where its presence is required to attenuate DAG signaling and initiate PA signaling^6^,^8^,^46^. Substitution of key residues at the SAM oligomer interface abolishes DGKδ oligomerization and constitutively localizes the enzyme to the plasma membrane^6^. Analogous to the role of DGKδ-SAM, SAM-mediated oligomerization of SMSr influences its subcellular localization. While an oligomerization competent form of SMSr predominantly localizes to the ER, mutations that destabilize SMSr oligomers cause a partial redistribution of the enzyme to the Golgi. Conversely, treatment of cells with curcumin, a drug that stabilizes SMSr homo-oligomers, promotes retention of the enzyme in the ER. It should be noted that combining three point mutations that each disrupt self-assembly of SMSr-SAM domains *in vitro* proved insufficient to completely abolish the formation of SMSr trimers and hexamers *in vivo*. This likely explains why removal of the SAM domain results in a more pronounced redistribution of the enzyme to the Golgi than introduction of mutations that only partially undermine SMSr homo-oligomerization. Together, our data indicate that SAM-mediated oligomerization of SMSr serves a critical role in retaining the enzyme in the ER. This is a remarkable finding in view of the fact that oligomerization has been frequently identified as a prerequisite for efficient export of membrane proteins from the ER^47^,^48^,^49^,^50^,^51^. How oligomerization of SMSr prevents the enzyme from entering COPII vesicles that bud from the ER remains to be established. One possibility is that SMSr carries an ER-export signal that is masked by its homotypic oligomerization. Alternatively, SMSr oligomers, in contrast to monomers, interact with an ER-resident protein to actively retain them in the ER or simply fail to enter COPII vesicles because of their size or shape.

SMSr belongs to the SMS enzyme family, which includes the Golgi-resident SM synthase SMS1. The latter enzyme also contains an *N*-terminal SAM domain and possesses the ability to self-associate. However, both SMS1 self-assembly and its localization to the Golgi complex were completely independent of its SAM domain. Moreover, the SMS1-SAM domain lacks the basic assembly of interface residues that are critical for homo-oligomerization of SMSr-SAM and DGKδ-SAM. Thus, even though SMSr and SMS1 display a substantial degree of structural similarity, their SAM domains appear to serve distinct roles. We previously showed that swapping the entire *N*-terminal cytosolic tail of SMS1 for that of SMSr blocked export of the chimera from the ER^21^. In contrast, exchanging only the SAM domain did not prevent the enzyme from reaching the Golgi. We anticipate that the activity of SMSr-SAM as ER retention signal in the SMS1 chimera is overruled by an ER-export signal present downstream of its SAM domain. In support of this notion, adding a *C*-terminal KKXX ER-retrieval signal failed to retain SMS1 in the ER but readily restored the ER-residency of SMSrΔSAM.

SMSr is a critical regulator of ER ceramides, at least in a variety of cultured mammalian cell lines^21^. The substantial rise in ER ceramides observed upon acute (siRNA-mediated) depletion of SMSr is difficult to account for by loss of the rather modest CPE-producing activity of the enzyme^21^. We recently reported that SMSr-catalyzed CPE production, although required, is not sufficient to suppress ceramide accumulation, ruling out metabolic conversion of ceramides as the primary mechanism by which SMSr controls ER ceramide levels^25^. Instead, we found that SMSr-mediated control over ER ceramides relies on its SAM domain; while removal of SAM did not affect SMSr-catalyzed CPE production, it abolished the ability of the enzyme to suppress ceramide accumulation even when SMSrΔSAM is retained in the ER^25^. This implies that SMSr-SAM, besides trapping the enzyme in the ER, directly participates in the mechanism by which SMSr controls ER ceramide levels. Application of bifunctional lipid analogues in photo-affinity labeling experiments with recombinant SMSr-SAM did not reveal any particular affinity of this domain for any of the enzyme’s lipid substrates or products. In addition, chemical crosslinking studies showed that catalytic activity of SMSr is dispensable for the ability of the enzyme to form homo-oligomers. Attempts to address whether fluctuations in ER ceramide levels influence the oligomeric state of SMSr are inconclusive. Our finding that curcumin stabilizes SMSr homo-oligomers most likely reflects a secondary effect of this compound as chemical crosslinker^44^ rather than its ability to stimulate *de novo* ceramide production in the ER^33^,^34^. Whether ER ceramides have an impact on SMSr oligomerization remains to be established. Dynamic changes in the stoichiometry of membrane proteins are hard to resolve by bulk biochemical approaches like chemical crosslinking or co-immunoprecipitation analysis. Therefore, our ongoing efforts focus on the application of single-molecule fluorescence microscopy as complementary method to monitor SMSr oligomerization in intact cells^52^.

## Methods

### Reagents

*N*-ethyl maleimide (NEM), dithiothreitol (DTT) and myriocin were from Sigma-Aldrich, 1,2-dioleoyl-sn-glycero-3-phosphocholine (DOPC), 1,2-dioleoyl-*sn*-glycero-3-phosphoethanol-amine (DOPE) and 1-palmitoyl-2-{6-[(7-nitro-2-1,3-benzoxadiazol-4-yl)amino]hexanoyl}-*sn*-glycero-3-phosphoethanolamine (NBD-PE) from Avanti Polar Lipids, and dithiobis(succinimidyl-propionate) (DSP), mouse monoclonal anti-HA agarose beads, C_6_-NBD-ceramide and N_3_-Alexa-Fluor647 from Thermo Fischer Scientific. Curcumin and thapsigargin were from Enzo Life Sciences, tamoxifen, fuminosin B1 and BAPTA-AM from Cayman, z-VAD-fmk from Merck Millipore, and L-arabinose from Carl Roth. Mouse monoclonal anti-V5 agarose beads were from Bethyl Laboratories and Ni-NTA agarose from Qiagen.

### Antibodies

The following antibodies were used: mouse monoclonal anti-β-actin (cat. no. A1978, 1:10,000; Sigma-Aldrich), rabbit polyclonal anti-FLAG (cat. no. 2368, 1:1000; Cell Signaling), mouse monoclonal anti-PARP-1 (sc8007, 1:1000; Santa Cruz), rabbit polyclonal anti-calnexin (sc11397, 1:1000; Santa Cruz), mouse monoclonal anti-V5 antibody (R960-25, 1:4000; Invitrogen), rat monoclonal anti-HA antibody (12158167001, 1:4000; Roche), rabbit polyclonal anti-HA antibody (71-5500, 1:1000 Invitrogen), goat polyclonal anti-calnexin (sc-6465, 1:200; Santa Cruz), sheep anti-TGN46 (AHP500, 1:200 AbD Serotec) and mouse monoclonal anti-GM130 antibody (610823, 1:200; BD Biosciences). Goat anti-mouse (cat. no. 31430, 1:5,000) and goat anti-rabbit IgG conjugated to horseradish peroxidase (31460, 1:5,000) were from Thermo Fischer Scientific. The following secondary antibodies were used for indirect immuno-fluorescence: FITC-conjugated donkey anti-sheep/goat (STAR88F, 1:200; AbD Serotec), Cy3-conjugated donkey anti-mouse IgG (715-165-150, 1:200; Jackson ImmunoResearch), Cy5-conjugated donkey anti-rabbit IgG (711-175-152, 1:200; Jackson ImmunoResearch), Cy2-conjugated donkey anti-mouse IgG (715-225-150, 1:200; Jackson ImmunoResearch), Cy5-conjugated donkey anti-goat IgG (705-175-147, 1:200; Jackson ImmunoResearch), Cy3-conjugated donkey anti-rabbit IgG (711-165-152, 1:200; Jackson ImmunoResearch).

### DNA constructs

scGFP-DGKδ-SAM, scGFP-DGKδ-SAM^V52E^, scGFP-SMSr-SAM and scGFP/pBAD bacterial expression vectors were kindly provided by James Bowie, University of California, Los Angeles, USA^29^. For mammalian expression of *C*-terminal V5/His6- or HA-tagged human SMSr and SMS1, the corresponding cDNAs were PCR amplified and cloned into pcDNA3.1/V5-His TOPO (Invitrogen) according to the manufacturer’s instructions. SAM-deficient truncation mutants SMSr∆SAM and SMS1∆SAM were obtained by deleting the first 78 and 68 *N*-terminal residues, respectively. The *C*-terminal sequence KKSA was added to retrieve V5/His6-tagged SMSr∆SAM in the ER. The first 68 *N*-terminal residues of HA-tagged SMS1 were replaced with the first 78 *N*-terminal residues of SMSr to generate SAMr-SMS1-HA. Mammalian expression vector pSEMS (Covalys Biosciences) containing monomeric eGFP (meGFP) is described in Wilmes *et al*.^53^. To obtain meGFP-SMSr, a cDNA encoding human SMSr was PCR amplified and inserted into pSEMS/meGFP via XhoI and NotI restriction sites. To obtain meGFP-SMSrΔSAM, the first 78 *N-*terminal amino acid residues of SMSr were removed during the PCR reaction. A mammalian expression construct encoding FLAG-tagged CERT was described in Tafesse *et al*.^25^ and a yeast expression construct encoding V5-tagged human SMSr-V5 in Kol *et al*.^31^. Single amino acid substitutions were introduced by site-directed mutagenesis using the megaprimer PCR method^54^.

### Native gel analysis

The scGFP constructs were transformed into *E. coli* TOP10 cells (Invitrogen) and transformants were inoculated in 100 ml LB medium containing 50 μg/ml ampicillin and grown to 0.6 OD_600_ with shaking at 37 °C. After addition of 0.2% L-arabinose, the cultures were transferred to 16 °C for 12–16 h, harvested by centrifugation and resuspended in 1 ml of 20 mM Tris pH 7.4, 1 M NaCl, 1 mM TCEP, 5 mM MgCl_2_, 5 mg/ml lysozyme, 20 μg/ml DNase I and protease inhibitor cocktail (1 μg/ml aprotinin, 1 μg/ml leupeptin, 1 μg/ml pepstatin, 5 μg/ml antipain, 157 μg/ml benzamidine) followed by 3 rounds of freeze-thawing and 3 rounds of sonication. The lysates were centrifuged at 20,000 × *g* for 30 min at 4 °C and the supernatant was used for native gel analysis. To this end, 37.5 μl supernatant was mixed with 12.5 μl 4× DNA/Native sample buffer (Expedeon) and loaded onto an Any kD™ Mini-PROTEAN^®^ TGX Stain-Free™ Protein Gel (BIO-RAD) after normalization of samples for total GFP fluorescence. The gel was run in 27.3 mM Tris-HCl and 192 mM glycine at 4 °C and visualized using a Typhoon FLA 9500 Biomolecular Imager (GE Healthcare) with 473 nm excitation laser and BPB1 emission filter (530/30).

### Lipid photoaffinity labeling

Synthesis of photoactivatable and clickable lipid (pacLipid) analogues is described in Supplementary Information. Liposomes containing specified pacLipids were prepared from a defined lipid mixture (DOPC/DOPE/pacLipid, 80/20/1 mol%) in CHCl_3_/MeOH (9/1, v/v) using a mini-extruder (Avanti Polar Lipids). In brief, 10 μmol of total lipid was dried in a Rotavap and the resulting lipid film was resuspended in 1 ml Buffer L (50 mM Tris-HCl, pH-7.4, 50 mM NaCl) by vigorous vortexing and sonication, yielding a 10 mM lipid suspension. Liposomes with an average diameter of ~100 nm were obtained by sequential extrusion of the lipid suspension through 0.4-micron, 0.2-micron and 0.1-micron track-etched polycarbonate membranes (Whatman-Nuclepore) and stored under N_2_ at 4 °C. Bacterially expressed scGFP-SAM fusion proteins (see above) were purified using Ni-NTA agarose chromatography according to instructions of the manufacturer, diluted to 40 μg/ml in Buffer L and mixed with pacLipid-containing liposomes at 1:1 (vol:vol). Samples were incubated at 37 °C for 1 h with shaking and then subjected to UV-irradiation using a 1000 W high-pressure mercury lamp (Oriel Photomax) equipped with a pyrex glass filter to remove wavelengths below 350 nm at a 30 cm distance from the light source. After addition of 10 μg soybean trypsin inhibitor as carrier protein, samples were subjected to CHCl_3_/MeOH precipitation and the resulting protein pellets were resuspended in PBS/1% SDS for 15 min at 70 °C. Click-reactions were performed by incubating ~20 μg of total protein per sample in 25 μl PBS/1% SDS containing 1 mM Tris(2-carboxyethyl)phosphin (TCEP), 0.1 mM Tris[(1-benzyl-1H-1,2,3-triazol-4-yl)methyl]amin) (TBTA), 1 mM CuSO_4_ and 80 μM N_3_-AlexaFluor647 for 1 h at 37 °C. Next, 5× Sample Buffer (300 mM Tris, pH 6.8, 10% (w/v) SDS, 50% (v/v) glycerol, 10% (v/v) β-mercaptoethanol and 0.025% (w/v) bromophenol blue) was added and samples were heated to 95 °C for 5 min prior to SDS-PAGE. The gel was fixed in 40% ethanol and 10% acetic acid at RT for 1 h, subjected to in-gel fluorescence analysis using a FLA-9500 Biomolecular Imager with 635 nm excitation laser and LPR emission filter (665LP), and then stained using Coomassie Blue.

### Cell transfection, chemical crosslinking and immuno-affinity chromatography

HeLa cells (ATCC-CCL2) were cultured in high-glucose DMEM supplemented 10% FBS. Cells were transfected with DNA constructs using Effectene (Qiagen) according to instructions of the manufacturer. For crosslinking experiments, cells were treated with 50 mM DSP in PBS for 15 min at RT and quenched with 25 mM Tris pH 7.4 for 10 min prior to lysis. At 24 h post-transfection, the cells were lysed in Lysis Buffer (1% TritonX-100, 1 mM EDTA pH 8.0, 150 mM NaCl, 20 mM Tris pH 7.5, 10 mM *N-*ethyl maleimide and protease inhibitor cocktail). Nuclei were removed by centrifugation at 15,000 × *g* for 10 min at 4 °C and post-nuclear supernatants were incubated with anti-HA, anti-V5 or Ni-NTA agarose beads for 3 h at 4 °C. Beads were washed extensively in Lysis Buffer and then in PBS at 4 °C. Affinity-purified proteins were eluted in 2% SDS at RT and then analysed by SDS-PAGE and immunoblotting. Generation of a SMSr-knockout HeLa cell line using CRISPR/Cas9 technology (HeLa SMSr^−/−^) was performed as described in Supplementary Information. SMSr^−/−^ cells were selected by immunoblotting using an affinity-purified rabbit polyclonal anti-SMSr antibody^22^ and CPE synthase activity assay as described in Vacaru *et al*.^21^.

### Yeast culture and CPE synthase activity assay

Yeast strain IAY11 (*MATα ade2-1 trp1-1can 1-1000 leu2-3 112 his3–11,15 ura3-52 ade3-∆853*) was transformed with pYES2.1/SMSr-V5-His TOPO expression constructs and grown in synthetic medium containing 2% (w/v) galactose to early mid-logarithmic phase. For crosslinking experiments, cells were treated with indicated amounts of DSP in PBS for 15 min and quenched with 25 mM Tris pH 7.4 for 10 min at RT prior to lysis. Cells were collected by centrifugation and lysed in ice-cold Buffer R (15 mM KCl, 5 mM NaCl, 20 mM HEPES/KOH pH 7.2, 10 mM NEM and protease inhibitor cocktail) by bead bashing. Nuclei were removed by centrifugation at 700 × *g* for 10 min at 4 °C. Post-nuclear supernatants (PNS) were subjected to quantitative immunoblot analysis or CPE synthase activity assay as in Ternes *et al*.^30^. Briefly, 40 nmol 1-palmitoyl-2-oleoyl-sn-glycero-3-phosphoethanolamine was dried under a stream of nitrogen. 200 μl Buffer R containing 0.002% Triton X-100 was added and sonicated in a water bath for 15 min. A total of 200 μl postnuclear supernatant was added and the mixture was incubated on ice for 1 h. The reaction was started by adding NBD-Cer from a 2 mM ethanolic stock to a final concentration of 25 μM. Enzyme reactions were incubated at 37 °C for 1 h with constant shaking. The reaction was stopped by adding 1 ml MeOH and 0.5 ml CHCl_3_. Phase separation was induced by adding 0.5 ml CHCl_3_ and 0.5 ml of 0.45% (w/v) NaCl. The lower phase was evaporated under a stream of nitrogen, dissolved in 25 μl CHCl_3_:MeOH (2:1) and then spotted on HP-TLC plates (Macherey & Nagel) using a CAMAG Linomat 5 TLC sampler. The TLC was developed first in acetone, dried, and then in CHCl_3_:MeOH:25%NH_4_OH (50:25:6, v-v:v). Fluorescent lipids were visualized using a Typhoon FLA 9500 Biomolecular Imager. NBD-labeled SMS reaction products were quantified using known amounts of NBD-PE as reference.

### Fluorescence microscopy

HeLa cells were seeded on glass coverslips and transfected with HA- or GFP-tagged SMS constructs using Effectene. After 24 h, cells were fixed with 4% (w/v) paraformaldehyde in PBS, quenched in 25 mM NH_4_ and permeabilized using PBS containing 0.1% (w/v) saponin and 0.2% (w/v) BSA. Coverslips were immunostained using primary and fluorophore-conjugated secondary antibodies essentially as in Vacaru *et al*.^21^. Coverslips were mounted using Prolong Gold Antifade Reagent (Molecular Probes). Images were captured using a confocal microscope (Olympus LSM FV1000) with two spectral and a single standard detector, a UPLSAPO 60x/NA 1.35 oil immersion objective (Olympus) and an Olympus laser box with AOTF laser combiner. GFP/FITC/Cy2 fluorophores were excited with a 488 nm Argon laser, Cy3 was excited with a 559 nm diode laser and Cy5 was excited with a 635 nm diode laser. Excitation light was reflected by a 405/488/559/635 nm dichroic mirror. Emitted light was collected using secondary dichroic mirrors SDM-560 and SDM-640 and a barrier filter BA 655–755 for GFP/FITC/Cy2, Cy3 and Cy5, respectively. To avoid crosstalk between imaging channels, images for different fluorophores were collected sequentially in a descending order of wavelength i.e. from long to short. Imaging of entire cells was done by acquisition of z series of 250 nm step size. Quantitation of Golgi-associated levels of GFP-tagged SMSr and calnexin was performed using ImageJ software (NIH). First, Golgi regions in transfected cells were determined using anti-GM130 immunostaining. Fluorescence intensity values from areas without any cells were taken as background values. After background subtraction, the ratio of GFP or calnexin signal intensity within the Golgi region (Manders’ coefficient) were determined using the JaCoP plugin of ImageJ software.

## Additional Information

**How to cite this article:** Cabukusta, B. *et al*. ER residency of the ceramide phosphoethanolamine synthase SMSr relies on homotypic oligomerization mediated by its SAM domain. *Sci. Rep.* **7**, 41290; doi: 10.1038/srep41290 (2017).

**Publisher's note:** Springer Nature remains neutral with regard to jurisdictional claims in published maps and institutional affiliations.

## Supplementary Material

Supplementary Information
