# Supplementary material for: ER residency of the ceramide phosphoethanolamine synthase SMSr relies on homotypic oligomerization mediated by its SAM domain
Source: Sci Rep. 2017 Jan 25;7:41290. doi: 10.1038/srep41290 (PMC5264588; doi:10.1038/srep41290)
Supplement: Supplementary Information [file srep41290-s1.pdf]

## SUPPLEMENTARY INFORMATION

### ER residency of the ceramide phosphoethanolamine synthase SMSr relies on homotypic oligomerization mediated by its SAM domain

Birol Cabukusta[1], Matthijs Kol[1], Laura Kneller[1], Angelika Hilderink[1], Andreas Bickert[2], John G.M. Mina[1], Sergei Korneev[1], Joost C.M. Holthuis[1*]

*[1]Molecular Cell Biology Division, Faculty of Biology/Chemistry, University of Osnabrück, 49076 Osnabrück, Germany: [2]Molecular Genetics, Life and Medical Sciences Institute, University of Bonn, 53115 Bonn, Germany*

[*]Correspondence to: Joost C.M. Holthuis, Molecular Cell Biology Division, University of Osnabrück, Barbarastrasse 13, 49076 Osnabrück, Germany; Phone: +49 541969 7140; Fax: +49 541969 2884; E-mail: holthuis@uos.de

**SUPPLEMENTARY METHODS**

***Synthesis of photoactivatable and clickable lipid analogues***

A 15 carbon-long fatty acid containing a photo-activatable diazerine and clickable alkyne group, pacFA, was synthesized in 3 steps from commercially available educts as described in Haberkant *et al*[1]. Next, pacFA was coupled to D-*erythro*-sphingosine (Enzo Biochem) using a combination of 1-ethyl-3-(3-dimethylaminopropyl)carbodiimide (EDCI) and hydroxybenzotriazole (HOBT) as condensing reagents, yielding the photo-activatable and clickable C15-ceramide analogue, pacCer (85% overall yield). pacPC was synthesized starting from 1-oleoyl-2-hydroxy-*sn*-glycero-3-phosphocholine (Avanti Polar Lipids) and pacFA under the action of N,N-dicyclohexylcarbodiimide (DCC) and 4-dimethylaminopyridine (DMAP) with satisfactory yield (39%). pacDAG was synthesied in 3 steps starting from 1-oleoyl-*sn*-glycerol (Santa Cruz Biotechnology). First, the primary HO-group was protected with the triphenylmethyl protecting group (trityl-chloride/pyridine; 92% overall yield). The glycerol obtained was coupled with the pacFA using EDCI/DMAP activation (58% overall yield). The final deprotection step was achieved using trifluoroacetic acid (TFAA) to generate pacDAG (28% overall yield). pacPE was synthesied in 3 steps starting from 1-oleoyl-2-hydroxy-*sn*-glycero-3-phosphoethanolamine (Avanti Polar Lipids). First, the amino-group was protected with the *tert*-butoxycarbonyl protecting group (di-*tert*-bytuldicarbonate/triethylamine; 98% overall yield). The ethanolamine obtained was coupled with pacFA using EDCI/DMAP activation in a good yield (52%). The final deprotection step was achieved with TFAA to generate pacPE (35%, overall yield). pacSM was synthesized starting from sphingosylphosphorylcholine (lyso-SM d18:1, Avanti Polar Lipids) and pacFA under the action of EDCI/HOBT (78% overall yield). pacCPE was synthesized in a single step from pacSM using a transphosphatidylation exchange reaction of choline fragment to ethanolamine initiated by phospholipase D Type VII from *Streptomyces sp*. (Sigma Aldrich), essentially as described in Fletcher *et al*[2]. All synthetic compounds were purified by thin layer chromatography to a high degree (purity >98%) and their structures were confirmed by 1H and 13C NMR and electrospray-ionisation mass spectrometry (ESI MS). The synthetis of photoactivatable and clickable lipid analogous will be described in detail in (S. Bockelmann, S. Korneev, J. Mina, P. Haberkant and J. Holthuis, manuscript in preparation).

***Generation of SMSr$^{-/-}$ cells***

Generation of a CRISPR/Cas9-mediated SMSr-knockout HeLa cell line (HeLa SMSr$^{-/-}$) was performed essentially as described Mali *et al.*[3]. Briefly, a gBlock containing the CRISPR target sequence was designed as follows:

cacagtcagacagtgactcaGTGTCACAgctagcTTTCCCATGATTCCTTCATATTTGCATATACGATACAAG
GCTGTTAGAGAGATAATTAGAATTAATTTGACTGTAAACACAAAGATATTAGTACAAAATACGTGA
CGTAGAAAGTAATAATTTCTTGGGTAGTTTGCAGTTTTAAAATTATGTTTTAAAATGGACTATCATA
TGCTTACCGTAACTTGAAAGTATTTCGATTTCTTGGCTTTATATATCTTGTGGAAAGGACGAAACA
CCG**TCAACTCTGCATTCGCCGC**GTTTTAGAGCTAGAAATAGCAAGTTAAAATAAGGCTAGTCCGT

TATCAACTTGAAAAAGTGGCACCGAGTCGGTGCTTTTTTTggatccTGTGCACAgtcagtcacagtcagtcta c - (CRISPR target sequence for SMSr in bold and underlined).

The gBlock was digested with NheI and BamHI and ligated into the NheI/BamHI sites of a CMV promoter-deleted pCDH-EF1-Hygro vector (re-named pCDH-CMV(-); SBI; CD515B-1). HeLa cells stably transfected with doxycycline-inducible Cas9 expression construct pCW-Cas9 (kindly provided by F.G. Tafesse, Oregon Health and Science University, Portland, Oregon) were transfected with pCDH-CMV(-)SMSr/sgRNA and cultured in media containing 7µg/ml puromycin and 250µg/ml hygromycin B (Invitrogen). Stably transfected cells were treated with 2µg/ml of doxycycline (Sigma-Aldrich) for 3-5 days. The cells were transferred to a 96-well plate at clonal dilution. Individual colonies were picked, and propagated. SMSr−/− cells were selected by immunoblotting using an affinity-purified rabbit polyclonal anti-SMSr antibody[4] and CPE synthase activity assay as described in Vacaru *et al.*[5].

## REFERENCES


1. Haberkant, P. *et al.* In vivo profiling and visualization of cellular protein-lipid interactions using bifunctional fatty acids. *Angew. Chem. Int. Ed. Engl.* **52,** 4033–8 (2013).
2. Fletcher, S., Ahmad, A., Price, W. S., Jorgensen, M. R. & Miller, A. D. Biophysical properties of CDAN/DOPE-analogue lipoplexes account for enhanced gene delivery. *ChemBioChem* **9,** 455–463 (2008).
3. Mali, P. *et al.* RNA-guided human genome engineering via Cas9. *Science* **339,** 823–826 (2013).
4. Bickert, A. *et al.* Functional characterization of enzymes catalyzing ceramide phosphoethanolamine biosynthesis in mice. *J. Lipid Res.* **56,** 821–835 (2015).
5. Vacaru, A. M. *et al.* Sphingomyelin synthase-related protein SMSr controls ceramide homeostasis in the ER. *J. Cell Biol.* **185,** 1013–27 (2009).

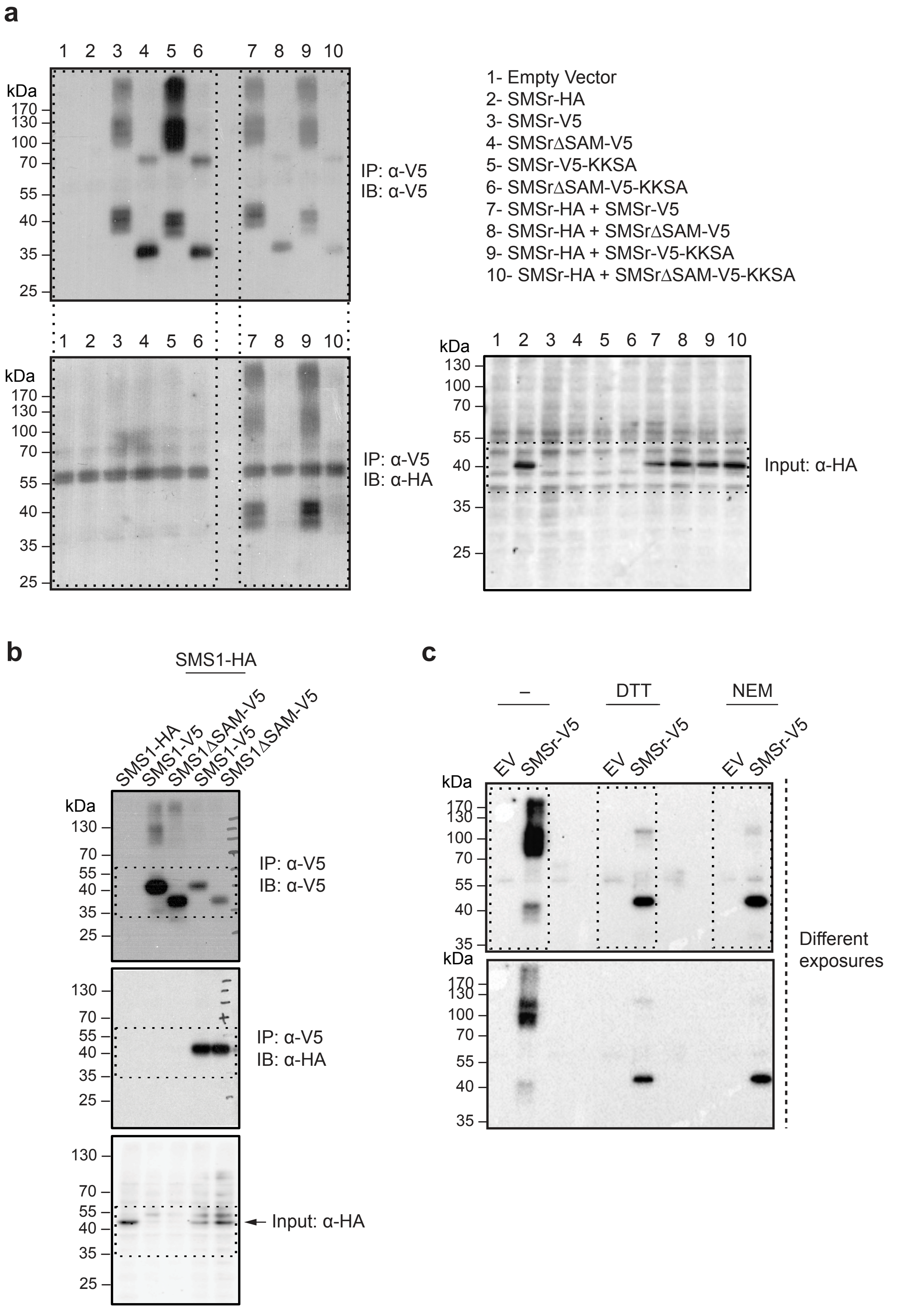


**Figure S1. Uncropped blots shown in Figure 3.** Uncropped images of blots used to make the corresponding panels in Fig. 3 are shown. Only parts of the blots delineated by dashed boxes were used. Other annotations are as in Fig. 3 and are described in its legend.

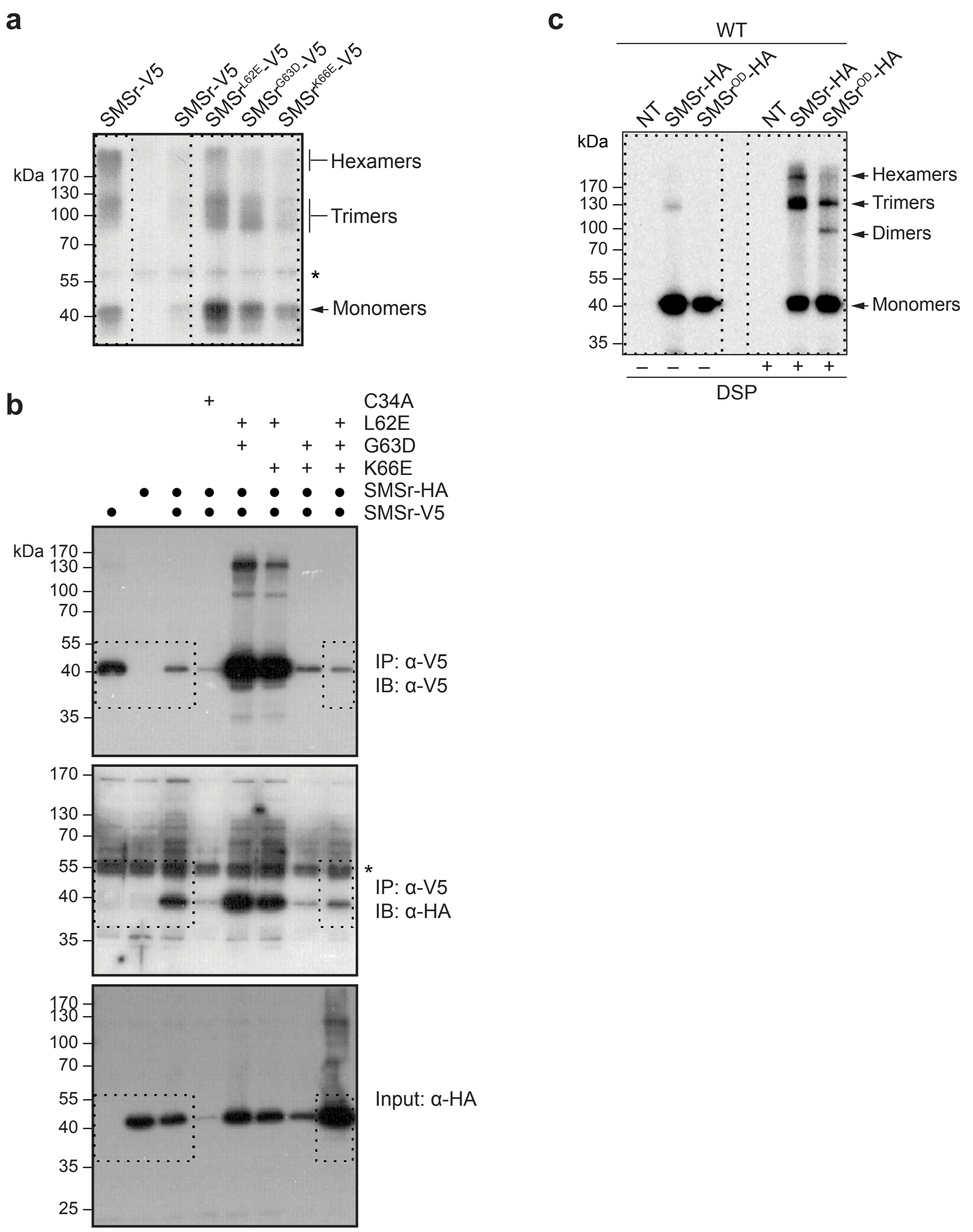


**Figure S2. Uncropped blots shown in Figure 4.** Uncropped images of blots used to make the corresponding panels in Fig. 4 are shown. Only parts of the blots delineated by dashed boxes were used. Other annotations are as in Fig. 4 and are described in its legend.

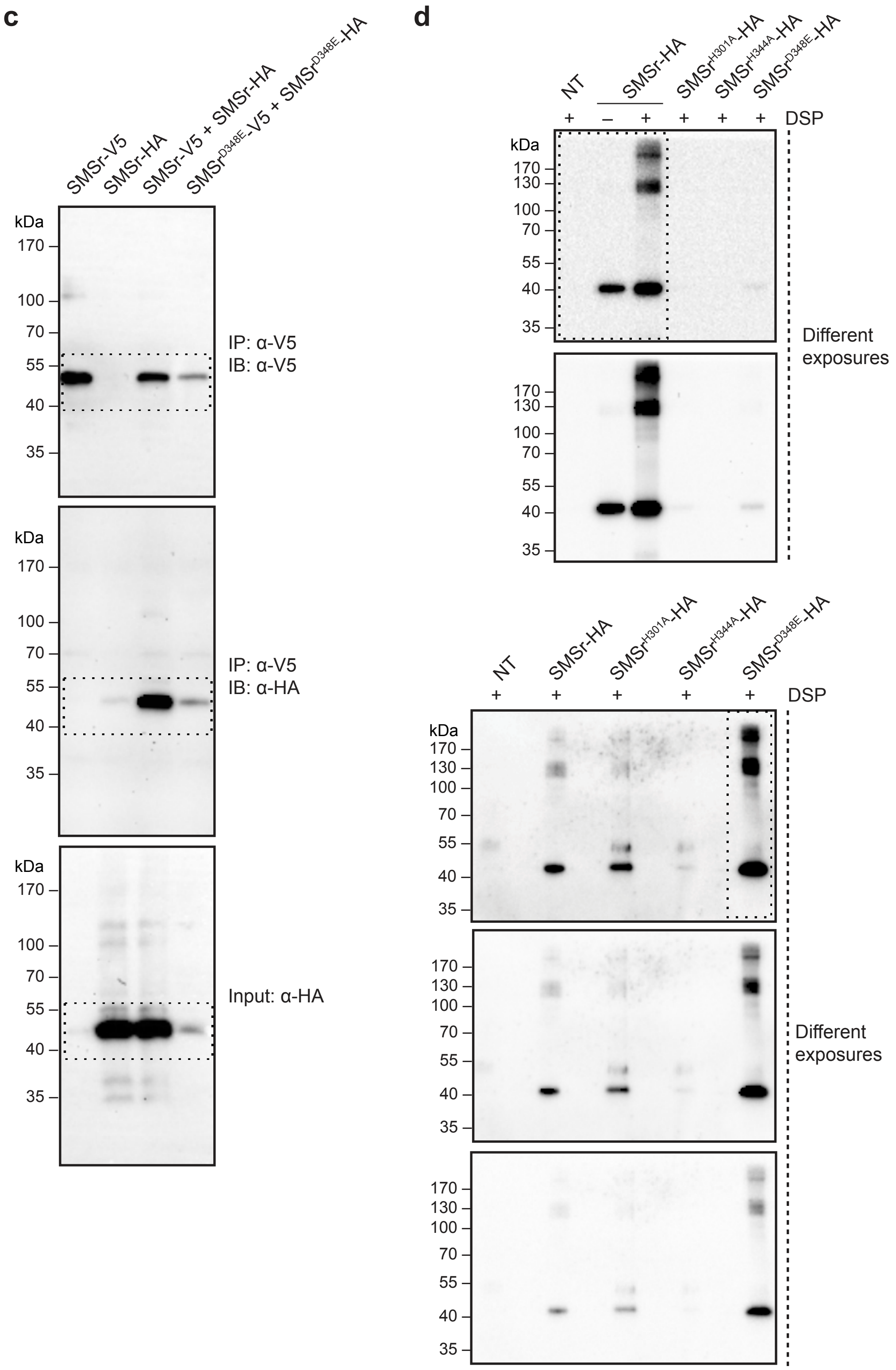


**Figure S3. Uncropped blots shown in Figure 5.** Uncropped images of blots used to make the corresponding panels in Fig. 5 are shown. Only parts of the blots delineated by dashed boxes were used. Other annotations are as in Fig. 5 and are described in its legend.

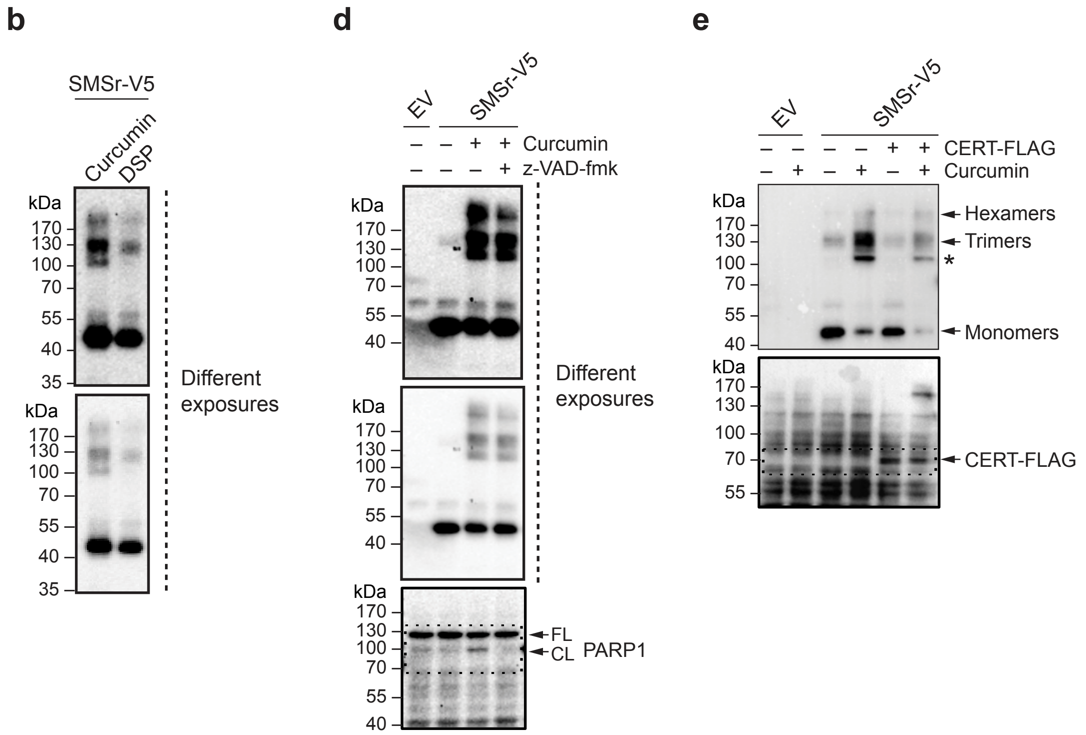


**Figure S4. Uncropped blots shown in Figure 6.** Uncropped images of blots used to make the corresponding panels in Fig. 6 are shown. Only parts of the blots delineated by dashed boxes were used. Other annotations are as in Fig. 6 and are described in its legend.

| Query: hSMSr-SAM | | | | | |
|---|---|---|---|---|---|
| *Accession* | *Description* | *Query cover* | *E value* | *Ident* | *Accession* |
| SAMD8 | Sphingomyelin synthase-related protein 1 | 100% | 2E-40 | 100% | Q96LT4.2 |
| DGKD | Diacylglycerol kinase delta | 98% | 3.E-05 | 33% | Q16760.4 |
| DGKH | Diacylglycerol kinase eta | 98% | 1.E-04 | 35% | Q86XP1.1 |
| LIPB2 | Liprin-beta-2 | 95% | 0.002 | 38% | Q8ND30.3 |
| CSKI1 | Caskin-1 | 100% | 0.006 | 36% | Q8WXD9.1 |
| SMS1 | Sphingomyelin synthase 1 | 68% | 0.02 | 37% | Q86VZ5.2 |
| BICC1 | Protein bicaudal C homolog 1 | 79% | 0.049 | 32% | Q9H694.2 |
| ETV6 | Transcription factor ETV6 | 94% | 0.1 | 35% | P41212.1 |
| LIPB1 | Liprin-beta-1 | 95% | 0.27 | 34% | Q86W92.2 |
| BFAR | Bifunctional apoptosis regulator | 100% | 0.58 | 25% | Q9NZS9.1 |

| Query: hDGKd-SAM | | | | | |
|---|---|---|---|---|---|
| *Accession* | *Description* | *Query cover* | *E value* | *Ident* | *Accession* |
| DGKD | Diacylglycerol kinase delta | 100% | 9.E-40 | 100% | Q16760.4 |
| DGKH | Diacylglycerol kinase eta | 100% | 1.E-30 | 83% | Q86XP1.1 |
| SHAN2 | SH3 and multiple ankyrin repeat domains protein 2 | 95% | 7.E-12 | 48% | Q9UPX8.3 |
| SHAN3 | SH3 and multiple ankyrin repeat domains protein 3 | 100% | 2.E-09 | 39% | Q9BYB0.3 |
| SHAN1 | SH3 and multiple ankyrin repeat domains protein 1 | 84% | 3.E-09 | 48% | Q9Y566.2 |
| CNKR3 | Connector enhancer of kinase suppressor of ras 3 | 85% | 1.E-07 | 37% | Q6P9H4.1 |
| CNKR2 | Connector enhancer of kinase suppressor of ras 2 | 85% | 5.E-07 | 37% | Q8WXI2.1 |
| SAM15 | Sterile alpha motif domain-containing protein 15 | 95% | 9.E-07 | 36% | Q9P1V8.1 |
| BICC1 | Protein bicaudal C homolog 1 | 81% | 1.E-05 | 37% | Q9H694.2 |
| SAMD8 | Sphingomyelin synthase-related protein 1 | 98% | 3.E-05 | 33% | Q96LT4.2 |

| Query: hSMS1-SAM | | | | | |
|---|---|---|---|---|---|
| *Accession* | *Description* | *Query cover* | *E value* | *Ident* | *Accession* |
| SMS1 | Sphingomyelin synthase 1 | 100% | 2E-41 | 100% | Q86VZ5.2 |
| UBIP1 | Upstream-binding protein 1 | 81% | 2.E-04 | 33% | Q9NZI7.1 |
| SAMD8 | Sphingomyelin synthase-related protein 1 | 67% | 2.E-04 | 37% | Q96LT4.2 |
| LIPB1 | Liprin-beta-1 | 62% | 6.E-04 | 31% | Q86W92.2 |
| CNKR3 | Connector enhancer of kinase suppressor of ras 3 | 73% | 0.002 | 27% | Q6P9H4.1 |
| TF2L1 | Transcription factor CP2-like protein 1 | 90% | 0.003 | 26% | Q9NZI6.1 |
| TFCP2 | Alpha-globin transcription factor CP2 | 56% | 0.003 | 31% | Q12800.2 |
| ICAM4 | Intercellular adhesion molecule 4 | 42% | 0.007 | 43% | Q14773.1 |
| CNKR2 | Connector enhancer of kinase suppressor of ras 2 | 71% | 0.008 | 26% | Q8WXI2.1 |
| SPDEF | SAM pointed domain-containing Ets transcription factor | 62% | 0.013 | 27% | O95238.1 |

**Supplementary Table 1. BLAST search reveals a high level of similarity between DGKδ-SAM and SMSr-SAM.** blastp searches (*http://blast.ncbi.nlm.nih.gov*) were performed in the swissprot database of human proteome. Queries sequences used are hSMSr-SAM, Q96LT4:12-78; hDGKδ-SAM, Q16760:1145-1208; hSMS1-SAM, Q86VZ5:13-76.

| Query | Accession | Residue # |
|---|---|---|
| human SMSr-SAM | NP_001167627.1 | 12-78 |
| mouse SMSr-SAM | NP_080559.1 | 75-141 |
| chicken SMSr-SAM | XP_426501.3 | 12-78 |
| frog SMSr-SAM | NP_001016197.1 | 11-77 |
| zebrafish SMSr-SAM | NP_001082939.1 | 12-78 |
| human DGKδ-SAM | NP_690618.2 | 1141-1205 |
| mouse DGKδ-SAM | NP_808314.2 | 1147-1211 |
| chicken DGKδ-SAM | XP_422569.4 | 1096-1160 |
| frog DGKδ-SAM | XP_004917921.1 | 1117-1181 |
| zebrafish DGKη-SAM | XP_005168036.1 | 1186-1249 |
| human SMS1-SAM | NP_671512.1 | 1-72 |
| mouse SMS1-SAM | NP_659041.3 | 7-78 |
| chicken SMS1-SAM | NP_989721.2 | 1-72 |
| frog SMS1-SAM | NP_001008197.1 | 1-72 |
| zebrafish SMS1-SAM | NP_001071082.1 | 1-72 |

**Supplementary Table 2. Accession numbers and amino acid residues used to create the phylogenetic tree in Figure 2c**
